# Profiling consumers with an environmentally sustainable and healthy diet: The case of Spanish households

**DOI:** 10.3389/fnut.2022.1035142

**Published:** 2022-11-10

**Authors:** Belén Gutiérrez-Villar, Rosa Melero-Bolaños, Maria Jose Montero-Simo, Rafael A. Araque-Padilla

**Affiliations:** Department of Management, Universidad Loyola Andalucía, Córdoba, Spain

**Keywords:** organic food, sustainable diet, NOVA classification, consumer profiles, Spain, mosaic plots

## Abstract

Our diet has substantial implications not only for our health but also for the environment. However, the two dimensions are not comparable, even though consumers often associate them with their purchasing choices. Promoting more sustainable diets requires a better knowledge of household profiles considering the healthy and organically sustainable character of the food purchased. Previous studies have approached the analysis of consumer profiles separately, differentiating both dimensions without clear conclusion regarding the variables that make up these profiles. In this study, we looked for household profiles by cross-referencing the organic nature of the products consumed (environmental sustainability) with their degree of processing (healthfulness) in Spain. The results show that the most sustainable products are consumed in tiny municipalities (less than 2,000 inhabitants). In contrast, less sustainable products are consumed in high-income, single-family households or households with small children. The person responsible for the purchase is working or between 39 and 45 years old. In conclusion, our study shows that socio-demographic variables are statistically significant in identifying household profiles with sustainable diets.

## Introduction

### Sustainable diet

Currently, the estimation of food consumption, nutrient intake, and food safety are growing in interest worldwide. So, in its latest published study, the Food and Agriculture Organization (FAO) stresses the urgency for the needed broader food systems transformation, which is currently the focus of global attention (1). Therefore, if the focus is on consumption, the need to raise awareness and promote a sustainable diet has been gaining ground. Diets are drivers of sustainable food systems (2) and inextricably link human health and environmental sustainability (3). So, a sustainable diet comprises different dimensions, such as health, ecological and social, all within the framework of political, economic, and environmental contexts that limit or facilitate changes in diets and food systems (4).

In this study, focused on the ecological and health dimensions of a sustainable diet, we will analyze the consumption of organic products, consider the degree of food processing, and identify consumer profiles for each case. All this is in a particular context, such as that of Spain. Organic production refers to the agricultural and animal husbandry management procedure of food production whose objectives ensure a viable and sustainable agricultural management system that combines production techniques aimed at the optimal and sustainable use of natural resources, reducing the environmental impact (5). In a more multifaceted conception, organic production combines tradition, innovation, and science to benefit the shared environment and promote fair relationships and good quality of life for all involved (6).

At the world level, organic production and consumption of organic food follow a growing trend (7, 8). A quick review of the evolution of the latest published data is shown in Table 1.

**TABLE 1:** Central figures on the production and consumption of organic food (8).

Indicator	2013	2019
Countries with organic production	170	187
Hectares dedicated to organic farming	35.1 million	72.3 million
Number of countries with organic regulations	82	108
Organic market size	54 billion euro	106.4 billion euro
Per capita consumption	7.03 euro	14.0 euro

An increasing number of consumers generally show positive perceptions of organic food products, understanding that they are healthier than their conventional counterparts and better for the environment (9, 10). There is a broad consensus that organic food production and consumption offer health and sustainability upsides (11–13).

In this context, sustainable diets protect and respect biodiversity and ecosystems, are culturally acceptable, accessible, economically fair and affordable, and nutritionally adequate, safe and healthy while improving natural and human resources (14). The issue has received considerable attention in Europe. From 2021, The European Council is pursuing the goal of transforming the way food is produced and consumed in Europe to reduce the environmental footprint of food systems and strengthen their resilience against crises.

Organic products are regulated by a series of regulations and standards by countries or areas. In this research, the definition of an organic product is in line with the description of European legislation. In 2007, the Council of the European Union (EU) agreed on Council Regulation 834/2007 (15), which establishes the principles, objectives, and general rules of organic production and defines how organic products should be labeled. Today, the Regulation in force is (EU) 2018/848 (16) on organic production and labeling of organic products. Specifically, an organic product is defined as a product resulting from organic production; organic production uses methods that comply with the Regulation at all stages of production, preparation, and distribution. The products of hunting or fishing wild animals are not considered organic products.

Specifically, the organic labels certify that a product complies with organic quality standards. So, consumers can be guided by this label when purchasing an organic product and ensure that the components comply with the organic standards. The EU organic logo can only be used on products containing at least 95% organic ingredients and respects other strict conditions for the remaining 5%. The same component cannot be present in organic and non-organic forms (17).

On the other hand, this research considers the classification of foods according to their degree of processing since many studies have evaluated their possible effect on consumer health. The bibliography shows that there are currently seven possible food classification systems based on the degree of processing. NOVA and SIGA are the two most globally applicable classification systems, i.e., they are not country or region-specific (18).

NOVA system has been used in most studies to analyze and document the effect of ultra-processed food consumption on various diseases or markers of disease, health, or mortality. Several cross-sectional and longitudinal studies have been conducted, with many indicating a direct relationship between increased consumption of ultra-processed foods and cardiovascular disease, obesity, type 2 diabetes, cancer, and generally a higher mortality risk (19–22).

Therefore, it is worth noting that NOVA has been used in nutrition research to assess the healthiness of diets and food environments where food composition data has not been readily available (23). It is the case we are dealing with in this paper. NOVA classifies all foods and food products into four groups according to their nature, the scope, and the purpose of the industrial processing to which they are subjected (24, 25). Currently, four groups are described (see Table 2), considering the physical, biological, and chemical processes to which the food is subjected before being consumed (26).

**TABLE 2:** Description of NOVA foods group (24–27).

Nova group	Description	Processes
Group 1 “Unprocessed or minimally processed foods”	Foods that may be consumed by themselves (such as fruits, nuts, milk, …); or as the main item or accompanying items of dishes and meals (such as grains, flours, vegetables, meat, eggs, …)	There are unprocessed foods altered by industrial processes such as removal of inedible or unwanted parts, drying, crushing, grinding, fractioning, roasting, boiling, pasteurization, refrigeration, freezing, placing in containers, vacuum packaging, or non-alcoholic fermentation. None of these processes adds salt, sugar, oils, or fats, or other food substances to the original food. Their main aim is to extend the life of grains (cereals), legumes (pulses), vegetables, fruits, nuts, milk, meat, and other foods, enabling storage for more extended use, and often making their preparation easier or more diverse
Group 2 “Processed culinary ingredients”	Food products obtained directly from group 1 foods or from nature by industrial processes such as pressing, centrifuging, refining, extracting, or mining (such as oils, butter, sugar, salt, …). Their use is in preparing, seasoning and cooking Group 1 foods	Processes such as pressing, centrifuging, refining, extracting, or mining
Group 3 “Processed foods”	These products are made by adding salt, oil, sugar, or other substances from group 2 to group 1	Processes such as canning and bottling, and, in the case of pieces of bread and cheeses, using non-alcoholic fermentation. Food processing here aims to increase the durability of group 1 foods and make them more enjoyable by modifying or enhancing their sensory qualities
Group 4 “Ultra-processed food and drink products”	Formulations of ingredients, most of exclusive industrial use that results from a series of industrial processes (hence “ultra-processed”), many requiring sophisticated equipment and technology	Processes enabling the manufacture of ultra-processed foods include the fractioning of whole foods into substances, chemical modifications of these substances, and assembly of unmodified and modified food substances. Ingredients characteristic of ultra-processed foods can be divided into food substances of no or rare culinary use and classes of additives whose function is to make the final product palatable or often hyper-palatable. These substances are rarely used in cooking and used only in the manufacture of ultra-processed foods, including varieties of sugars (fructose, high-fructose corn syrup, “fruit juice concentrates,” inverted sugar, maltodextrin, dextrose, lactose), modified oils (hydrogenated or interesterified oils), and protein sources (hydrolyzed proteins, soya protein isolate, gluten, casein, whey protein and “mechanically separated”). Cosmetic additives, also used only in manufacturing ultra-processed foods, are flavors, flavor enhancers, colors, emulsifiers, emulsifying salts, sweeteners, thickeners, and anti-foaming, bulking, carbonating, foaming, gelling, and glazing agents. These additives disguise undesirable sensory properties created by ingredients, processes or packaging used to manufacture ultra-processed foods or give the final product sensory properties especially attractive to see, taste, smell, or touch

It should be noted that there are foods with organic certification in any of the four NOVA categories, and not only in NOVA 1 of unprocessed or minimally processed foods. For example, organic oils (NOVA 2), organic wines (NOVA 3), organic frozen pizzas (NOVA 4), and other possible processed or ultra-processed foods are marketed in the Spanish market.

So, not all products labeled as organics are healthy, as may be the case with some ultra-processed foods. For example, are ultra-processed products-that tortillas chip or candy- that we can buy with European organic labeling if 95% of their agricultural ingredients are of organic origin.

As regards the country studied, it should be pointed out that Spain is a Mediterranean country. The Mediterranean diet is characterized by high consumption of olive oil, vegetable products, fish and seafood; low consumption of dairy, meat and meat products; and a moderate ethanol intake (28).

### Consumer profiles of organic food

As organic food grows in popularity, there is a growing need for academic research on consumer profiles, habits, and attitudes toward organic food (29). Previous research has addressed the relationship between organic food and sustainable consumption (30). That is, to understand organic consumption as a desire to be more sustainable. People are attracted to organic food because it is considered safe, healthy, and environmentally friendly (31), which impacts their daily-consumer preferences (32–34). However, although many studies show that organic food consumption is growing worldwide, there are still inconsistencies arising from differences in how people perceive organic food, their motivations, attitudes, or willingness to purchase (30, 35). These inconsistencies reveal a gap between intentions and attitudes at the center of behavioral research in this field (9, 36–38). Aspects such as price (39–44) and, to a lesser extent, availability and lack of knowledge and information (45–49) can be a barrier to buying organic products. Nevertheless, understanding that gap is made difficult by variations in consumer profiles. In previous research papers, the socio-demographic variables studied to profile consumers of organic products are gender, age, income, marital status, the existence of children in the household, education, and place of residence. The results are not conclusive. So, discussion of the pertinence of these variables for distinguishing between consumers of organic food and other consumers has not always enjoyed general agreement. There are studies suggesting no significant correlation between socio-demographic variables and purchase. Others offer a significant and negative correlation, while others have found a significant and positive correlation. A review of the literature on the different socio-demographic factors that impact the purchase of organic food is summarized in Table 3.

**TABLE 3:** Impact of socio-demographic factors on the purchase/purchase intention of organic foods.

Variables	Research result	References
Gender	Higher consumption or purchase intention among women	(32, 34, 38, 50–57)
	Higher consumption or purchase intention among men	(58)
	Not statistically significant	(46, 59–62)
Age	Positively correlated (higher consumption or intention to purchase among older people)	(35, 50, 63)
	Negatively correlated (higher consumption or intention to purchase among young people)	(61, 62)
	Not statistically significant	(46, 51, 59, 60)
Incomes/social class	Positively correlated (higher consumption or purchase intention with higher incomes)	(38, 46, 50, 51, 63–65)
	Negatively correlated (higher consumption or purchase intention with lower incomes)	(66)
	Not statistically significant	(61)
Employment status	Positively correlated (working or working in more qualified jobs)	(67)
	Negatively correlated	(66)
Education level	Positively correlated (higher consumption or purchase intention with higher education)	(38, 49, 52, 61, 63, 67, 68)
	Negatively correlated (higher consumption or purchase intention with lower education)	(61)
	Not statistically significant	(50, 69)
Children in the household	Positively correlated	(50, 51, 70, 71)
	Negatively correlated	(66)

These studies indicate a relationship between socio-demographic variables and the consumption of organic products. However, the findings reveal that there is no identified prototype profile of buyers of organic products.

### Objective and research question

To promote a sustainable diet, it is essential to know who is buying organic products and what kind of organic products they are buying, given that the previous studies reviewed do not yield conclusive results on the characteristics of organic food consumers. A set of sequential research questions has been designed, not assuming *a priori* that there are differences in who buys and what is eaten. So, are three questions: (1) to explore whether there are statistically significant differences in the socio-demographic profile of Spanish households that buy organic products and those that do not; (2) to investigate whether there are statistically significant differences in the degree of processing of organic and non-organic foods purchased by the households; (3) to discover the association between socio-demographic characteristics and the purchase of organic products in the four categories NOVA of processed and unprocessed foods. The weight justifies the choice of Spain as the object of research that its organic production represents worldwide: the third place on the world podium of the largest organic agriculture producers in 2019, only surpassed by Austria and Argentina in the ranking. In terms of consumption, it is in tenth place, with the USA, Germany and France being the most prominent organic food consumers (8).

## Materials and methods

### Data source

In the subject at hand, food purchases can be obtained mainly through different procedures, depending on the consumption unit studied in the data collection -household or individual-, the methodology used in the collection of information -stable sample or not over time, automatic or manual data collection-, the desired level of statistical significance -random or convenience sampling- and the nature of the entity that performs them -public agencies or private companies-. Fundamentally, the consumption data of a population can be obtained through panels or surveys carried out by public or private organizations, on households or individuals, chosen randomly or not, who undertake to collect all purchases made by manually recording them in purchase diaries or capturing the data using automated procedures.

In the case of Spain, the two primary official sources that collect food purchases were reviewed: the National Household Budget Survey and the National Household Food Consumption Panel. Although the first of these sources is available at the micro-data level, it could not be used because it does not measure organic food consumption. In contrast, the Household Consumption Panel of the Ministry of Consumption, Fisheries and Food has started to count the purchase of some categories of organic food in 2021 (72).

Another option, such as purchasing data from private organizations, has been discarded due to a lack of budget. The use of data from different sources has not been contemplated since some recent studies have detected that using different methodologies in data analysis may complicate or impair the combination of data from various sources (73).

The Household Food Consumption Panel (purchasing panel) is included in the National Statistical Plan. It represents all households in Spain and is statistically significant at the national level. The information is collected through a sample of 12,500 households, which are asked to record all their daily food purchases. Purchases are recorded daily using an optical barcode reader, which minimizes reporting errors. Food and beverages purchased to supply the household are collected every day of the year, at all times of purchase and by any household member. Products that do not have a barcode are collected by employing a code book that allows all types of products to be declared automatically. Self-consumption is not included (72). The composition of the average household basket in Spain, aggregating the data from the purchasing panel, is shown in Table 4.

**TABLE 4:** The composition of the average household basket in Spain in 2020.

Category	Volume share (%)
Total dairy products	17.1
Total fresh fruit	14.4
Total fresh vegetables	9.1
Total meat	7.0
Bread	4.7
Total potatoes	4.6
Total fish	3.6
Beer	3.4
Prepared dishes	2.6
Pastries-Cookies-Cereals	2.2
Total processed fruits and vegetables	2.0
Total vegetable oils	1.8
Total eggs	1.4
Rest of food	26.1

The official statistics present the information disaggregated according to the following socio-demographic criteria: the age of the person responsible for the expenditure, size of the municipality where the household is located; social class by income; the presence of children in the home by age; employment situation of the person responsible for purchasing; the number of persons in the household; household life cycle and autonomous territories. In this article, we have not studied information about the autonomous region of ascription (we were only interested in Spain as a whole). Neither has it been used family life cycle, as it is a variable that combines the age of the person responsible for purchasing, the number of family members, and the presence of children in the household. Unfortunately, it should be noted that the official Spanish statistics do not provide information on gender and education level.

Regarding the environment, the data on household consumption of organic products was incorporated for the first time in January 2020, so only one year’s information is available. On the other hand, data are only available for 19 organic food categories without disaggregating into subcategories. In this study, we have started from the information published and revised for 2020, choosing data measuring quantities purchased–kilograms. The numerical data available from this official statistic are totals−they can only be obtained in aggregate form in Excel files−it is not possible to get individualized micro-data by household.

Thus, the information necessary for this work has been extracted in the following way:

• Out of a total of 45 food categories collected by the household consumption panel, 19 categories containing organic food were selected;
• The data of the 19 similar non-organic food categories have been selected.

Household consumptions from official data are total, not per capita. Since this paper aims to analyze the differences between organic and non-organic consumption, the data from national statistics have been kept, not transformed into an average per person. However, the number of household members is one of the characteristics analyzed.

The 19 food categories, provided by official statistics, were assigned to the appropriate groups of NOVA. This assignment was done independently by two authors (BG and RM). Discrepancies between the two were recorded, and a final selection was agreed upon through consultation.

Hence, for the subsequent analyses, the kilograms of food purchased per household in Nova families have been added, which has shortened an initial list of 19 to only four groups. The quantities have been reduced to hundreds of thousands of kilos to facilitate the readability of the results (Table 5).

**TABLE 5:** Ninteen food categories-with organic and non-organic consumption data available for Spain in 2020-classified according to NOVA.

NOVA group	Food categories	Total consumption (organic and non-organic). Hundreds of thousands of kgs.	Percentage
NOVA 1 “Unprocessed or minimally processed foods”	Eggs; Meat; Milk; Rice; Pasta; Legumes; Potatoes; Vegetables; Fruits; Coffee, and infusions.	159,076	73.8
NOVA 2 “Processed culinary ingredients”	Oil; Flours.	7,705	3.6
NOVA 3 “Processed foods”	Bread; Wine; Juices; Nuts; Processed fruit, and vegetables.	31,794	14.8
NOVA 4 “Ultra-processed food and drink products”	Pastries; Ready meal	16,913	7.8

### Statistical methods

The use of contingency tables is the most common technique to study the relationships between variables taken in pairs. In this paper, we have chosen to analyze the aggregate data available according to the mosaic plots instead of presenting only contingency tables.

Mosaic plots, proposed by Hartigan and Kleiner (73, 74) and further developed (75), can help visualize contingency tables, even those complex ones consisting of many categorical variables. This kind of display can be beneficial in understanding simple and complex associations among categorical variables. So, the contingency tables can (but need not) be easy to understand simply by scanning the numbers (76), but this need not be the case with more complex tables, especially when the variables are associated, as is our case. In such cases, mosaic plots (or a fourfold visualization for 2 × 2 tables with an additional stratum variable) can help to capture associations between categorical variables.

To better understand the results presented in the following section, it is helpful to note that a mosaic plot is a graphical display of the cell frequencies of a contingency table in which the area of boxes of the plot is proportional to the cell frequencies of the contingency table. Both the size and position of the rectangles are relevant to the interpretation of the mosaic chart, making them one of the most advanced charts (77). For investigation, the mosaic function results in a mosaic plot in which the shading of the cells represents the Pearson residual for independence: red for negative values, blue for positive values, and more substantial shading for higher absolute residual values.

Analyses have been combined and performed sequentially: first, we checked, using contingency tables and the associated chi-square statistic, the existence of a one-to-one relationship between each socio-economic variable of the households and the purchase or not of organic products; subsequently, the distribution of food consumption according to NOVA was explored using mosaic plots and Pearson’s *p*-values of the residuals. Finally, cross-checking of socio-demographic categories with NOVA categories was conducted to detect possible differences in the case of organic NOVA products.

All analyses were carried out using R software.

It has not been possible to investigate multivariate relationships. Although we contacted the public body that publishes this information, obtaining the micro-data from the consumer panel was impossible. Only aggregated data were available, so applying multivariate analysis methods was impossible.

## Results

### Relationship between socio-demographic characteristics of Spanish households and the purchase of organic/non-organic foods

The analysis of the differences in the consumption of organic and non-organic foods was always significant; statistically significant differences were found for the six socio-demographic characteristics studied in Spanish households (*p* = 0,000 in each case). A closer inspection of the results, variable to variable, shows:

• More organic food is purchased than expected in households where the person responsible for the purchase does not work. No differences are observed in the case of non-organic products.
• Positive association with age: purchase of organic products higher than expected in the case of people aged 64 years or older; lower quantity purchased than desired in age groups under 49 years. Similar differences are seen in the opposite direction in the case of non-organic products.
• Negatively correlated with social class: higher organic food consumption with lower social classes.
• It was negatively correlated with the number of children: higher organic food consumption without children. In the case of children under 6 years of age, there is a higher-than-expected consumption of non-organic products in the household.
• The relationship is more diffuse for the number of people in the household; in the case of organic food, only families with two members consume more than expected, while non-organic food is more than expected in households with three members.
• Finally, regarding the size of the municipality, there is a negative correlation that shows a higher consumption of organic food in households located in municipalities with a smaller number of inhabitants.

Conclusively, the findings are statistically significant and show differences in the socio-demographic characteristics of the households buying organic food, at least in some aspects of the six variables studied.

### Differences between purchases of organic and non-organic foods according to their level of processing

**FIGURE 1:**
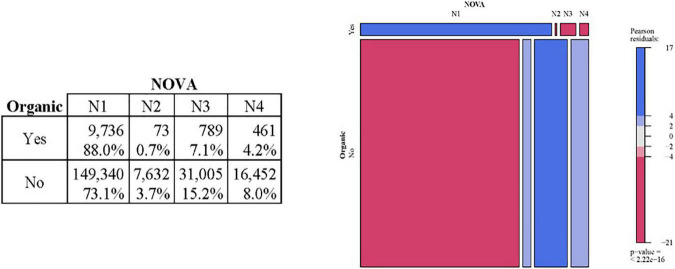
Relationship between the consumption of organic food and NOVA.

As a next step, it is helpful to examine whether there is a relationship between organic food consumption and the group consumed according to the Nova classification. Figure 1 contains the distribution of consumption according to both criteria, in absolute figures and percentages, taking in the second case as a reference the type of consumption (organic or not) and the mosaic plot with residual-based shading (organic × NOVA).

Calculating Pearson’s statistic, its *p*-value of 0.000 indicates that these characteristics are related so that the distributions of consumption by the NOVA group are not similar in the case of organic and non-organic products.

The differences found are as follows:

• In organic consumption, products from group NOVA 1 (unprocessed or minimally processed foods) account for 88.0%, significantly higher than the 73.1% non-organic consumption.
• The results are reversed for the other three groups (containing foods from less to more processed), and the percentages consumed among organic products are lower than in the non-organic case. The most significant difference occurs for NOVA 3 products, whose consumption is slightly more than double (7.1 vs. 15.2%).

### Relationship between socio-demographic characteristics of Spanish households and the purchase of organic products classified by NOVA

**FIGURE 2:**
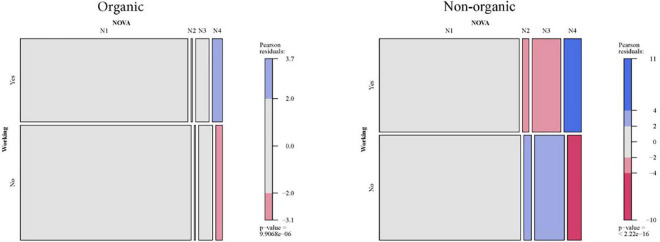
Relationship between employment situation of the person responsible for the household and NOVA: Organic vs. non-organic food purchases.

**FIGURE 3:**
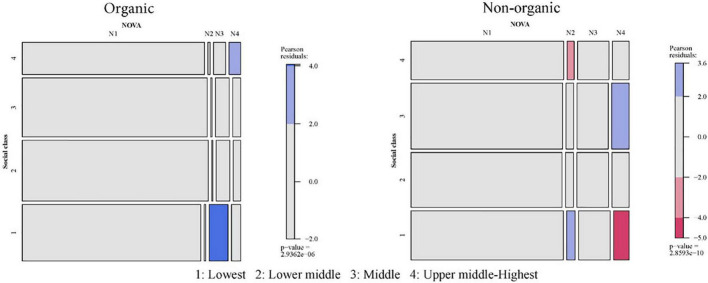
Relationship between social class by incomes and NOVA: Organic vs. non-organic food purchases. 1: Lowest; 2: Lower middle; 3: Middle; 4: Upper middle-highest.

**FIGURE 4:**
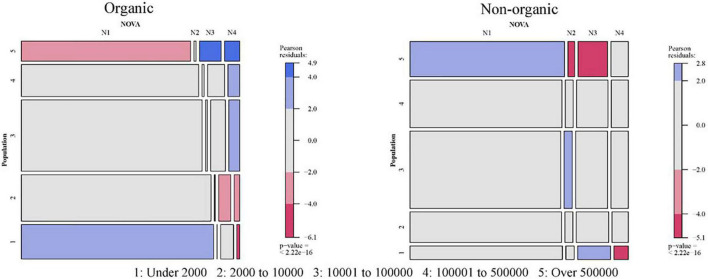
Relationship between size of the municipality and NOVA: Organic vs. non-organic food purchases. 1: Under 2,000; 2: 2,000–10,000; 3: 10,001–100,000; 4: 100,001–500,000; 5: Over 500,000.

**FIGURE 5:**
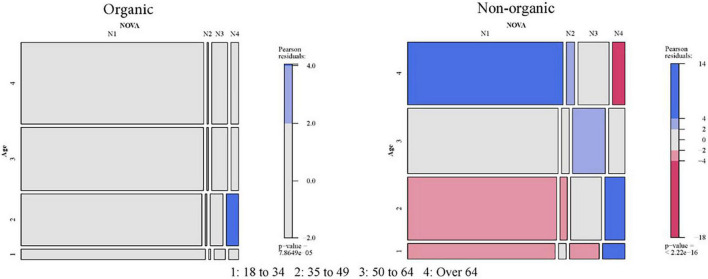
Relationship between the age of the person responsible for household buys and NOVA: Organic vs. non-organic food purchases. 1: 18–34; 2: 35–49; 3: 50–64; 4: Over 64.

**FIGURE 6:**
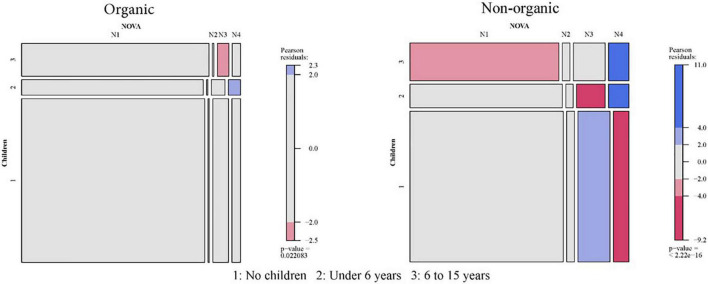
Relationship between the presence of children in the household and NOVA: Organic vs. non-organic food purchases. 1: No children; 2: Under 6 years; 3: 6–15 years.

**FIGURE 7:**
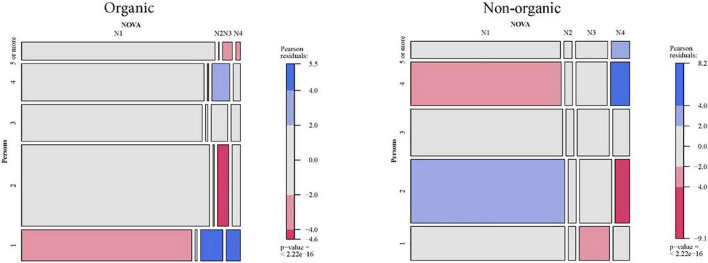
Relationship between the number of persons in the household and NOVA: Organic vs. non-organic food purchases.

The results will be extracted by comparing two mosaic plots associated with each of the contingency tables of the combination “socio-demographic characteristic × organic or non-organic × NOVA.” The six Mosaic plots for NOVA and socio-demographic characteristics are presented in Figures 2–7.

There are a few differences in the NOVA classification for the spectrum of organic foods, segmented according to each of the six socio-demographic variables available. However, quite a few differences are observed when the analysis is compared with Spanish households’ consumption of non-organic food. Therefore, the following paragraphs show the results for each organic category.

#### Organic NOVA 1 (Unprocessed or minimally processed foods)

Higher than-expected consumption has been found in the case of households located in tiny municipalities (less than 2,000 inhabitants). On the other hand, the deviation is negative for larger cities (more than 500,000 inhabitants). For the other variables, the consumption of this type of food is lower than expected only for one-person households.

#### Organic NOVA 2 (Processed culinary ingredients)

No differences exist in the NOVA 2 category when organic foods are analyzed.

#### Organic NOVA 3 (Processed foods)

NOVA 3 organic food consumption shows more statistical differences when crossing its consumption with the variable number of people in the household. So, one-person families, followed by four-person families, have higher than expected consumption of processed organic products. On the other hand, two-person households and households with five or more people have lower consumption.

In addition, the size of the municipality is also statistically significant, with higher consumption in the larger municipalities (more than 50,000 inhabitants) and lower-than-expected consumption in smaller cities (2,000 to 10,000 inhabitants).

Finally, the lower social class households also show higher consumption than expected.

#### Organic NOVA 4 (Ultra-processed foods)

NOVA 4 organic food consumption shows statistical differences when crossing its consumption with the six socio-demographic variables. However, it is interesting to note that more categories show higher than expected consumption of these ultra-processed organic foods.

The results show the following socio-demographic characteristics with higher than expected quantities of NOVA 4 purchased: responsible for the purchase working, aged 39–45 years, in one-person households, located in municipalities of 10,000 inhabitants or more, with high incomes. Slightly higher consumption is also detected in the case of the presence of children under 6 years of age.

On the other hand, consumption of ultra-processed organic products in lower-than-expected quantities has only been detected in cases where the person in charge of the household does not work or in households located in tiny municipalities (less than 10,000 inhabitants).

Finally, Table 6 summarizes the main results obtained by food group and socio-demographic characteristics.

**TABLE 6:** Summary of results of NOVA organic food crosses with socio-demographic variable.

Category	Organic food	Category	Organic food
NOVA 1	Higher consumption than expected: ● Households are located in tiny municipalities (less than 2,000 inhabitants).	NOVA 2	No statistically significant differences have been found.
	Lower consumption than expected: ● Larger municipalities (more than 500,000 inhabitants). ● One person households.		
	Not statistically significant: ● Age of the person responsible for the purchase. ● Employment situation of the person responsible for purchasing. ● Existence of children in the households. ● Social class.		
NOVA 3	Higher consumption than expected: ● One person or four persons in the households. ● Larger municipalities (more than 500,000 inhabitants). ● Low-income households.	NOVA 4	Higher consumption than expected: ● Person responsible for the purchase is working. ● Responsible for purchases in 39−45 age group. ● Medium and larger municipalities. ● High incomes. ● One person in the household. ● Children under 6 years old.
	Lower consumption than expected: ● Two or five persons in the households. ● Smaller municipalities (2,000 to 10,000 inhabitants).		Lower consumption than expected: ● Responsible for purchases is not working. ● Small municipalities (under 10,000 inhabitants).
	Not statistically significant: ● Age of the person responsible for the purchase. ● Employment situation of the person responsible for purchasing. ● Existence of children in the households.		

## Discussion

This research can help decision-makers associated with health and organic food production understand the socio-demographic factors related to purchasing organic food in households. Organic and healthy are not always synonymous and, as has been reflected in this work, official statistics add within organic some less healthy products, belonging to the categories of processed or ultra-processed foods (NOVA 3 and NOVA 4).

Also, another relevant contribution of this study has been not to assume *a priori* the existence of differences in the profiles of organic food buyers, depending on the basket of NOVA foods they purchase. The results show differences in the proportion of healthier foods among organic foods, with a higher-than-expected percentage of unprocessed foods (NOVA 1) and lower portions of foods purchased with some processing (NOVA 2, NOVA 3, and NOVA 4).

Although Spanish households have different consumption in the four NOVA groups, disaggregation by socio-demographic characteristics does not always leave conclusive results. Consistent with the literature, this research found that socio-demographic variables are statistically significant in identifying the profiles of organic food consumers. The findings are similar, for some socio-demographic categories of the variables, to those obtained in other investigations (35, 50, 63) and show evidence of higher consumption of organic products among older people. However, the current study’s findings do not support all the previous results, which established a positive relationship between employment status, income level, or the presence of children in the household with the consumption of organic food. In this research, not working or having lower incomes provide higher consumption, like the results found in work with Spanish data (66). Other findings not contrasted with previous studies show higher organic food consumption in small municipalities. The variable related to household size only gives differences in organic food consumption in the case of two-person households.

Lastly, the results relating to the third research question associate households consuming healthier diets with socio-demographic variables. This study shows that most rural households are the most likely to consume unprocessed organic foods (Organic NOVA 1). Moreover, suppose we study it from a complementary point of view. In that case, those responsible for households that consume less than expected ultra-processed foods are associated with low social class, unemployed, in small municipalities, under 50 years old, with a family structure consisting of the parents or, in the case of existing children are, under 6 years old.

Policymakers can use these results to design actions to promote the consumption of sustainable diets. In Mediterranean countries, such as Spain, at the forefront of organic farming worldwide, the results show higher consumption in rural municipalities with less than 2,000 inhabitants. The proximity to farms can be a determining factor in the shopping basket for healthy food, basically of category NOVA 1.

The age of the consumers is also behind this result. Smaller locations have an older population and have found that older people spend the most on unprocessed foods. This result is consistent with the ANIBES Survey (78)–conducted by the Spanish Nutrition Foundation–and would allow us to complete that within the NOVA 1 foods, the differences are occurring in the consumption of fruits. Thus, according to this study, in all age groups of the Spanish population, the most consumed food was milk and dairy products, followed by vegetables. However, among older people (65−75 years), the most consumed group was fruit, followed by milk and dairy products. In addition, the consumption of vegetables was higher in adults and older people than in the child and adolescent population; the elderly group was the only one with significantly higher consumption of this food group. Adults and the elderly than in the child and adolescent population; the elderly group was the only one with significantly higher consumption of this food group.

The results of this study, which show more traditional Mediterranean dietary patterns among older rural people, hint at recognizing a certain “westernization” in the dietary habits of younger people from Mediterranean countries who live in urban areas, as has already been shown in other previous studies (28, 79, 80).

Given the results obtained, it would be helpful for public authorities to promote the benefits of following the Mediterranean diet patterns; it would be a “successful formula” to achieve the double dimension of a sustainable diet: on the one hand, it is healthy (81) -it has been associated with a lower risk of developing cardiovascular disease and cardiometabolic risk factors, cancer, diabetes, neurodegenerative diseases and premature mortality or prevention of cardiovascular disease- and, on the other hand, it is more eco-friendly as it has a lower environmental impact, mainly due to its consumption of more plant-derived products and fewer animal products (82, 83).

As less sustainable dietary patterns are found in the youngest, schools could be fundamental in educating future consumers to follow Mediterranean nutritional habits, as dietary behavior patterns are acquired mainly at an early age (84, 85).

Therefore, if the aim is to promote sustainable diets in households that are not regular purchasers, it is worthwhile to promote very focused actions which, from the public authorities, seek to encourage the consumption of a sustainable diet in diverse ways for two different target groups: with more explicit and more eye-catching and smart labeling that highlights the concept of food suitable for a sustainable diet, with communication campaigns that bring “the farm” closer to the shoppers in the larger municipalities, with price promotions on sustainable food, of the organic food of NOVA 1 group; and perhaps most notably by reaching children, in an environment that encourages sustainable behavioral patterns that they will maintain into adulthood, for example, by implementing healthy menus in school canteens and contacting them with messages delivered through social media with the support of influencers whom these children and teenagers like the parents of the future. In this line, it will be interesting to see how the European Green Deal (86) will be developed and what results will be obtained, whose package of strategies includes measures to promote more sustainable diets.

This study has attempted to relate two aspects contributing to achieving a sustainable diet: its environmental and health components. The available data have not allowed us to develop the results obtained in more detail.

An explicit limitation of this research derives from the available sources of information. In the case of Spain, the first attempt was made to extract data from the Household Budget Survey, which, although not specifically designed for food, collects information on the consumption of food groups at the household level. An engrossing project carried out with data from this survey is the Proyecto European Food Availability Databank based on Household Budget Surveys (DAFNE), which has allowed essential conclusion to be drawn at the per capita level on food consumption patterns at the household level (87, 88). Unfortunately, this source of information could not be used in this research because the data collected lacks a distinction between organic and inorganic foods, so the environmental dimension of sustainable diets cannot be analyzed.

The main limitations of this study are the quality and quantity of data available. After locating information on the consumption of some organic foods in the Spanish Household Consumption Panel, data was again unavailable. Since we did not have individualized consumption data (per household), it was impossible to perform multivariate analyses and obtain the per capita consumption values. In addition, since this is a very recent statistic on organic foods, there are still very few varieties of food for which information is collected in Spanish households. We want to stress the importance for public administrations to make all available consumption data available to researchers and not only tables of aggregated values.

The other limitation of the study can be attributed to the measurement of the healthy dimension with the NOVA system. Although it is widely used, further clarification and justification of the NOVA classification would be desirable (87, 89).

We also support the need to standardize instruments for measuring population dietary patterns across countries, which would allow robustness of the results by making comparisons between different locations (90). Further work could include the design of statistical procedures and dashboards to combine food composition databases with household purchase panels.

Finally, we would like to emphasize again the need to collect more data on organic food consumption, which would allow us to have more extended and complete data series to estimate trends and make more accurate policies.

## Data availability statement

Publicly available datasets were analyzed in this study. This data can be found here: https://www.mapa.gob.es/es/alimentacion/temas/consumo-tendencias/panel-de-consumo-alimentario/series-anuales/.

## Author contributions

BG-V drafted the manuscript and prepared the figures and tables with the assistance and collaboration of RA-P, MM-S, and RM-B. All authors contributed to the development of the manuscript, conceptualization of this research, writing, and editing and read and agreed to the published version of the manuscript.
